# Expression and prognostic value of HER-2/neu in primary breast cancer with sentinel lymph node metastasis

**DOI:** 10.1042/BSR20170121

**Published:** 2017-08-02

**Authors:** Zhen-Jun Tong, Ning-Yao Shi, Zhi-Ji Zhang, Xiao-Dong Yuan, Xiao-Ming Hong

**Affiliations:** 1 Department of Thyroid and Breast Surgery, Ningbo Yinzhou No. 2 Hospital, Ningbo 315040, P.R. China; 2 Department of Cardiothoracic Surgery, Ningbo Yinzhou No. 2 Hospital, Ningbo 315040, P.R. China; 3 Department of General Surgery, Ningbo Yinzhou No. 2 Hospital, Ningbo 315040, P.R. China

**Keywords:** breast cancer, disease-free survival, human epidermal growth factor receptor-2, overall survival, prognosis, sentinel lymph node metastasis

## Abstract

The present study explores the correlation of human epidermal growth factor receptor-2 (HER-2) protein expression with sentinel lymph node (SLN) metastasis and prognosis of breast cancer. The breast cancer tissues and adjacent tissues were obtained from patients with primary breast cancer. Quantitative real-time polymerase chain reaction (qRT-PCR) was performed to detect the mRNA level of HER-2. Spearman correlation analysis was used to analyze the correlation of HER-2 expression with SLN metastasis. The disease-free survival (DFS) and overall survival (OS) of breast cancer patients were investigated. Univariate and multivariate analyses were performed to explore factors influencing SLN metastasis and prognosis of breast cancer. Compared with adjacent tissues, HER-2 expression was significantly up-regulated in breast cancer tissues. HER-2 expression was correlated with the pathological type, tumor node metastasis (TNM) staging, histological grade, blood vessel invasion, SLN metastasis, estrogen receptor (ER), and progesterone receptor (PR). The expression level of HER-2 was positively related to the SLN metastasis (*r*=0.548). Median DFS and OS were longer in patients with negative HER-2 expression than in patients with positive HER-2 expression. TNM staging, SLN metastasis, and expression levels of HER-2 and ER were independent factors for DFS of breast cancer patients, while TNM staging, blood vessel invasion, histological grade, SLN metastasis, and expression levels of HER-2 and PR were independent factors for OS of breast cancer patients. Our study suggests that high expression of HER-2 promoted SLN metastasis. HER-2 expression and SLN metastasis were the independent factors for the prognosis of breast cancer.

## Introduction

As the most common malignancy and the primary cause of tumor death in women around the world, breast cancer accounts for 25% of overall cancer cases and 15% female cancer deaths [1]. The incidence and mortality of breast cancer differ very remarkably in different countries and regions, with the highest rates in Europe and North America but the lowest rates in Asia [2]. Age, ethnicity, use of acyeterion, a family history, reproductive and endocrine factors, hormone therapy, obesity, excessive drinking, smoking, and lack of physical exercise have all been identified as the potential risk factors for breast cancer [3]. Over the past few years, the application of preventive measures, the early detection and diagnosis, and timely treatment for breast cancer have contributed to the significant reduction in death [4]. The sentinel lymph node biopsy (SLNB) is a highly sensitive, accurate, and secure way of diagnosing early breast cancer and predicting the metastasis status of breast cancer [5]. At present, the main treatments of breast cancer include surgery, radiotherapy and chemotherapy, and hormonal therapy [6,7]. Due to the complex pathogeny of breast cancer, more and more attention is being paid to the prognosis of patients with breast cancer, particularly from the perspective of molecular biomarkers [8].

Human epidermal growth factor receptor-2 (HER-2), also designated as HER-2/neu, c-erbB2, or ErbB2, is a 185 kDa transmembrane oncoprotein consisting of three distinct parts: an extracellular ligand-binding domain (ECD), a single α-helix transmembrane domain, and an intracellular tyrosine kinase domain [9]. HER-2 is a member of epidermal growth factor receptors (EGFRs) family, which is composed of four structurally similar proteins: HER1, HER2, HER3, and HER4 [10]. This receptor has a close correlation with cellular growth, apoptosis, proliferation, differentiation, angiogenesis, and aggression by regulating various signaling pathways [11]. It has been proved that *HER-2* gene amplification and protein overexpression play an essential role in the development of many types of solid tumors, such as breast, gastric, uterine cervix, bladder, and esophageal cancers [12–14]. In addition, the overexpression of HER-2 is directly linked with higher invasion and worse prognostic effects in breast, gastric, prostate, and other kinds of tumors [15,16]. Although there were many studies exploring the association of HER-2 with the detection, diagnosis, and prognosis of breast cancer, only a few researches focused on the relationship between HER-2 and SLN metastasis and prognosis in breast cancer. The present study is conducted to investigate the correlation of HER-2 protein expression with SLN metastasis and prognosis in breast cancer so as to test if HER-2 is a potential marker in sentinel lymph node (SLN) metastasis and prognosis of breast cancer.

## Materials and methods

### Ethical statement

The present study was approved by the Ethical Committee of Ningbo Yinzhou No. 2 Hospital. All patients have a good understanding of the present study and signed written informed consents.

### Study subjects

From January 2010 to June 2014, 316 female patients with primary breast cancer undergoing radical mastectomy or modified radical mastectomy in the Department of Surgical Oncology of Ningbo Yinzhou No. 2 Hospital were enrolled in the present study. All patients were confirmed pathologically and had not received any medication, radiotherapy, and chemotherapy before surgery. Their medical records and other related materials were complete. In addition, all patients had neither tumors in other parts of body nor a history of combined heart, liver, kidney, and other system diseases. The age of selected patients was 27–78 years old, with a mean age of 48.6 ± 15.3 years old. According to the tumor node metastasis (TNM) staging developed by the Union for International Cancer Control (UICC) in 2003 [17], there were 54 patients in stage I, 151 patients in stage II, 64 patients in stage III, and 47 patients in stage IV. Based on Scarff–Bloom–Richardson (SBR) grading [18], the histological grade was classified into three levels: grade I (highly differentiated) (*n*=37), grade II (moderately differentiated) (*n*=163), and grade III (lowly differentiated) (*n*=116). In accordance with the criteria of 2001 Chinese Pathological Classification of Tumors, the histopathological classification of breast cancer was divided into 31 invasive patients, 17 noninvasive patients, and 268 nonspecific invasive patients.

### Specimen collection

The tumor tissues of breast cancer and the surrounding area within 5 cm of tumor tissues were regarded as the cancer area, namely the breast cancer tissues. The normal breast tissues that were approximately 3–5 cm of the cancer area were selected as the adjacent tissues of breast cancer, which were confirmed to be absent of atypia by pathological sections. The breast cancer tissues and corresponding adjacent tissues of all included patients were obtained. All pathological specimens were fixed with formalin, embedded with paraffin, and sliced into 4 μm serial sections prepared for further use.

### Quantitative real-time polymerase chain reaction

For the quantitative real-time polymerase chain reaction (qRT-PCR) analysis, total RNA was isolated from the sections using miRNeasy micro kit (QIAGEN, GmbH, Germany). PrimeSeriPt@RT reagent Kit (Perfect Real Time) (TaKaRa, Tokyo, Japan) was applied for reverse transcription of total RNA to cDNA. The conditions of reverse transcription were as follows: 15 min at 37°C, and 5 s at 85°C. The samples were reserved at −20°C (the extracted procedure was similar to the vector construction). ABI PRISM 7500 real-time PCR System (ABI Company, Oyster Bay, NY, U.S.A.) was used for qRT-PCR amplification. PCR reaction conditions: predenaturation at 94°C for 5 min, followed by 35 cycles of denaturation at 94°C for 45s, annealing at 58°C for 45s, and extension at 72°C for 1 min. PCR reaction system (25 μl) was performed as follows, 10× PCR Buffer (2.5 μl), 25 mmol/l MgCl_2_ (1.5 μl), 10 mmol/l dNTP (0.5 μl), 10 mmol/l Primer (1 μl/l), 1 nmol/l Probe (2.5 μl/l), 5 μl/l, Taq (0.25 μl), cDNA (2.5 μl), sterile distilled water (15 μl). Glyceraldehyde-3-phosphate dehydrogenase (GAPDH) served as an internal reference. The primer sequences were shown in Table 1. Relative quantification was calculated using the 2^−^^ΔΔ*C*^_t_ method.

**Table 1:** Primer sequences of HER-2 and GAPDH for quantitative real-time polymerase chain reaction

Gene	Primer sequence
*HER-2*	5′-GCCCTCATCCACCATAACACC-3′
	5′-CATTCCTCCACGCACTCCTG-3′
*GAPDH*	5′-TGGTCTACATGTTCCAGTACT-3′
	5′-CCATTTGATGTTAGCGGGATCTC-3′

### Immunohistochemistry

The protein levels of estrogen receptor (ER), progesterone receptor (PR), and HER-2 were measured with streptavidin–peroxidase (SP) kit (Batch number: SP-9000; Beijing Zhongshan Biotechnology Co., Ltd, Beijing, China). The tissue sections were dehydrated with dimethylbenzene and gradient alcohol, retrieved with citrate antigen retrieval buffer of pH 7.2–7.4, and washed with phosphate buffer solution (PBS) for three times. Blocked with 5% albumin bovine serum (BSA) for 20 min, the tissue sections were subsequently stored at 4°C overnight with the addition of 50 μl of mouse anti-human HER-2 monoclonal antibody (dilution, 1: 500, Wuhan Boster Biological Technology Co., Ltd, Wuhan, China), followed by washing three times with PBS. And then second antibody, goat anti-mouse IgG (Wuhan Boster Biological Technology Co., Ltd, Wuhan, China), was added for the incubation at 37°C for 20 min. The sections were developed with diaminobenzidine (DAB) and counterstained with hematoxylin. After dehydration with gradient alcohol and mounted in neutral gum, the sections were finally observed under the light microscope. The positive HER-2 cells were mainly evaluated by cell membrane staining. The assessment contained four levels: (–) represented no membrane coloration, (+) indicated weak and incomplete membrane coloration, (++) meant complete but uneven brown membrane coloration of over 10% cells ranging from weak to moderate strength, or strong and complete brown membrane coloration of less than 30% cells, and (+++) represented strong and complete brown membrane coloration of over 30% cells. HER-2 was considered as positive with a level of (+++) and negative with a level of (–), (+), or (++) [19].

### Determination of SLN metastasis

SLN of breast cancer patients undergoing radical mastectomy or modified radical mastectomy was obtained. Then 2 ml of 1% Patent Blue was diluted to 4 ml and injected into upper, lower, left, and right areas around the lump. Fifteen minutes after injection, the skin flap was freed in routine method and blue-stained lymph vessel was subsequently identified. The blue-stained lymph node nearest to tumor was recognized as SLN, which was removed for further inspection. All SLN tissues were embedded in paraffin and sliced into normal sections. There were two sections for each SLN, one for hematoxylin–eosin (HE) staining and the other for immunohistochemistry (IHC) staining performed with mouse anti-human CK19 antibody. The selected SLNs were examined by the methods of routine pathological section (1–2 sections cut along the maximal diameter of lump and HE staining) and CK19 IHC. The SLN metastasis was determined as positive only when both results were positive. The interval serial section method (slice interval: 500 μm) was applied if CK19 IHC was positive and pathological section was negative, followed by the performance of routine HE staining for the second time. The SLN metastasis was classified into the positive expression if the staining was positive, otherwise it was regarded as the negative expression (remove of false positive) [20].

### Follow-up

A 48-month follow-up was conducted on all patients by June 2016. The duration was defined as the time from the first day of surgery until the date of death, loss to follow-up or the last time of follow-up. During this period, patients died of other causes, lost to follow-up or still alive by the end of follow-up were all recorded as censored. Those who were lost to follow-up were processed by recording the last time of follow-up. Follow-up information was collected by means of outpatient service, telephone, or medical record review. The survival time was calculated on the basis of month. Disease-free survival (DFS) referred to the period between the first day after surgery and the time when recurrence or metastasis occurred for the first time. Overall survival (OS) began from the first day after surgery and ended at the time of death or the last time of follow-up.

### Statistical analysis

Statistical analysis was carried out using SPSS 21.0 software (SPSS, Chicago, U.S.A.). Measurement data were displayed as mean ± standard deviation (SD) ($\bar{x}$ ± s), in which the comparison between two groups of normally distributed measurement data was analyzed by *t*-test. Enumeration data were expressed as percentage or rate and tested by chi-square test. The correlation was determined by Spearman correlation analysis. Kaplan–Meier survival curve was used for univariate analysis and Log-rank test was adopted for survival curve analysis. Cox proportional hazard regression model was applied for multivariate analysis. *P*<0.05 was considered as statistically significant.

## Results

### HER-2 expression in breast cancer tissues and adjacent tissues

**Figure 1:**
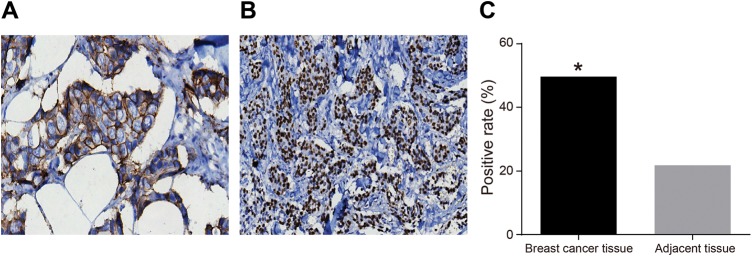
HER-2 expression in breast cancer tissues and adjacent tissues (SP ×200) (**A**) HER-2 expression in breast cancer tissues detected by SP. (**B**) HER-2 expression in adjacent tissue detected by SP. (**C**) The histogram of positive rate of HER-2 in breast cancer tissues and adjacent tissues. *P<0.05, compared with the adjacent tissues.

HER-2 expression in breast cancer tissues and adjacent tissues was detected by SP. The results indicated that weak and incomplete cell membrane coloration was observed in adjacent tissues, while strong and complete brown cell membrane coloration was found in breast cancer tissues (Figure 1A and B). The statistical analysis demonstrated that the positive expression rate of HER-2 was significantly up-regulated in breast cancer tissues compared with adjacent tissues (*P*<0.05) (Figure 1C), which indicated a higher level of HER-2 expression in breast cancer tissues.

### Associations of HER-2 expression with clinicopathological features of breast cancer

The analysis of HER-2 protein level and clinicopathological features of breast cancer demonstrated that HER-2 expression and HER-2 mRNA level were not associated with the age, menstrual condition, and tumor size of patients with breast cancer (all *P*>0.05), but was correlated with the pathological type, TNM staging, histological grade, blood vessel invasion, SLN metastasis, and expression levels of ER and PR (all *P*<0.05) (Table 2). Spearman correlation analysis was used to determine the correlation of HER-2 expression, HER-2 mRNA level, and expression levels of ER and PR with SLN metastasis. The results revealed that the positive rate of HER-2 was higher in patients with SLN metastasis than in patients without SLN metastasis, and that HER-2 expression and HER-2 mRNA level were positively correlated with SLN metastasis (*r*=0.548, *P*<0.001; *r*=0.221, *P*<0.001), while the expression levels of ER and PR were negatively related to SLN metastasis (*r* = −0.279, *P*<0.001;* r* = −0.293, *P*<0.001) .

**Table 2:** Correlations of HER-2 expression with clinicopathological features of breast cancer

Clinicopathological feature	Case	HER-2 expression		*χ*^2^	*P*	HER-2 mRNA	*χ*^2^	*P*
		Positive	Negative					
Age (year)				0.76	0.383			
≤36	106	48	58			0.57 ± 0.20	0.786	0.432
>36	210	106	104			0.59 ± 0.22		
Menstrual condition				1.344	0.246			
Premenopausal	201	93	108			0.60 ± 0.20	1.618	0.107
Menopausal	115	61	54			0.56 ± 0.23		
Tumor size (cm)				3.169	0.075			
≤2	71	28	43			0.59 ± 0.23	0.346	0.73
>2	245	126	119			0.58 ± 0.21		
Pathological type				10.27	0.006			
Noninvasive	17	2	15			0.44 ± 0.20	6.661	0.002
Invasive	31	14	17			0.51 ± 0.22		
Nonspecific invasive	268	138	130			0.60 ± 0.21		
TNM staging				60.23	<0.001			
Stage I	54	41	13			0.53 ± 0.22		
Stage II	151	43	108			0.57 ± 0.22	2.698	0.046
Stage III	64	32	32			0.62 ± 0.22		
Stage IV	47	38	9			0.63 ± 0.17		
Histological grade				6.083	0.048			
Grade I	37	17	20			0.50 ± 0.19	8.019	<0.001
Grade II	163	70	93			0.56 ± 0.21		
Grade III	116	67	49			0.64 ± 0.22		
Blood vessel invasion				22.93	<0.001			
No	277	121	156			0.55 ± 0.20	8.968	<0.001
Yes	39	33	6			0.82 ± 0.16		
SLN metastasis				41.23	<0.001			
No	109	26	83			0.52 ± 0.17	4.138	<0.001
Yes	207	128	79			0.62 ± 0.22		
ER				12.08	0.001			
Positive	198	79	114			0.56 ± 0.23	2.353	0.019
Negative	118	75	48			0.62 ± 0.20		
PR				5.389	0.02			
Positive	193	84	109			0.55 ± 0.19	3.289	0.001
Negative	123	70	53			0.63 ± 0.24		

### Correlations of HER-2 expression and SLN metastasis with survival of patients with breast cancer

**Figure 2:**
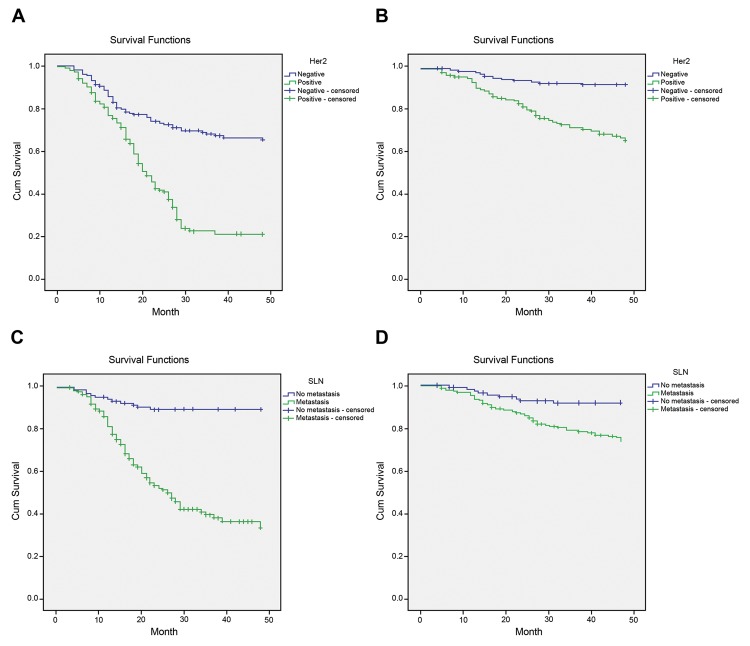
Kaplan–Meier survival curves of patients with different HER-2 expression and SLN metastasis (**A**) DFS survival curve of HER-2, with *P*<0.05 examined by Log-rank test. (**B**) OS survival curve of HER-2, with *P*<0.05 examined by Log-rank test. (**C**) DFS survival curve of SLN metastasis, with *P*<0.05 examined by Log-rank test. (**D**) OS survival curve of SLN metastasis, with *P*<0.05 examined by Log-rank test.

From the first day of surgery to June 2016, all patients were followed up to investigate their survival time, among which 26 patients who lost to follow-up were processed by recording the last time of follow-up. Kaplan–Meier survival curve indicated that the median DFS and OS of patients with negative HER-2 expression were 33.3 and 45.8 months respectively, but those of patients with positive HER-2 expression were 21.2 and 39.8 months respectively, and that the median DFS and OS of patients without SLN metastasis were 45.8 and 45.4 months respectively, but those of patients with SLN metastasis were 18.8 and 41.6 months respectively. Log-rank test demonstrated that median DFS and OS were significantly longer in patients with negative HER-2 expression than in patients with positive HER-2 expression, and that median DFS and OS were notably longer in patients without SLN metastasis than in patients with SLN metastasis (all *P*<0.05) (Figure 2).

### Univariate analysis of SLN metastasis and prognosis of patients with breast cancer

The clinicopathological features of breast cancer patients, including age, menstrual condition, tumor size, pathological type, TNM staging, histological grade, blood vessel invasion, SLN metastasis, and HER-2 expression, were included in univariate analysis. The results pointed out that tumor size, TNM staging, histological grade, and expression levels of HER-2, ER, and PR were factors for SLN metastasis (all *P*<0.05). It was also found that TNM staging, histological grade, blood vessel invasion, SLN metastasis, and expression levels of HER-2, ER, and PR would all affect DFS and OS of patients with breast cancer (all *P*<0.05). In addition, tumor size was the factor for DFS of patients with breast cancer (Table 3).

**Table 3:** Univariate analysis of DFS and OS with breast cancer

Risk factor	SLN metastasis		DFS		OS	
	RR (95% CI)	*P*	RR (95% CI)	*P*	RR (95% CI)	*P*
Age	1.005 (0.990–1.020)	0.525	1.009 (0.994–1.024)	0.266	1.003 (0.985–1.021)	0.765
Menstrual condition	0.923 (0.571–1.492)	0.743	1.110 (0.691–1.783)	0.665	1.018 (0.569–1.820)	0.953
Tumor size	2.579 (1.362–4.883)	0.004	1.827 (1.069–3.112)	0.028	1.399 (0.685–2.856)	0.357
Pathological type	2.003 (0.979–4.098)	0.057	0.667 (0.360–1.235)	0.198	0.930 (0.425–2.037)	0.857
TNM staging	1.947 (1.178–3.219)	0.009	1.980 (1.208–3.246)	0.007	4.322 (2.392–7.809)	<0.001
Histological grade	3.050 (1.786–5.211)	<0.001	2.448 (1.522–3.937)	<0.001	3.182 (1.582–6.399)	0.001
Blood vessel invasion	1.350 (0.677–2.695)	0.394	2.473 (1.304-4.668)	0.006	2.771 (1.391–5.520)	0.004
HER-2 expression	5.121 (3.105–8.444)	<0.001	2.052 (1.265–3.330)	0.004	3.915 (1.949–7.865)	<0.001
SLN metastasis	–	–	9.171 (4.645–18.106)	<0.001	3.728 (1.759–7.899)	0.001
ER	0.265 (0.154–0.456)	<0.001	0.571 (0.358–0.912)	0.019	0.399 (0.209–0.761)	0.005
PR	0.289 (0.171–0.488)	<0.001	0.160 (0.097–0.265)	<0.001	0.534 (0.292–0.977)	0.042

Abbreviations: CI, confidence interval; RR, risk ratio; –, no value.

### Multivariate analysis of SLN metastasis and prognosis of patients with breast cancer

Logistic regression model was used to analyze multiple factors that were shown to exert an effect on SLN metastasis in univariate analysis. The results manifested that tumor size, TNM staging, histological grade, and expression levels of HER-2, ER, and PR were independent factors for SLN metastasis of breast cancer (all *P*<0.05). Cox proportional hazard regression model was applied to analyze factors that were proved to affect the prognosis of breast cancer in univariate analysis. It was suggested that TNM staging, SLN metastasis, and expression levels of HER-2 and PR were independent factors affecting the DFS of patients with breast cancer (all *P*<0.05), and that TNM staging, blood vessel invasion, histological grade, SLN metastasis, and ER expression level were independent factors influencing the OS of patients with breast cancer (Table 4).

**Table 4:** Multivariate analysis of SLN metastasis and prognosis of patients with breast cancer

Risk factor	SLN metastasis		DFS		OS	
	RR (95% CI)	*P*	RR (95% CI)	*P*	RR (95% CI)	*P*
Tumor size	0.685 (0.327–1.437)	0.317	1.014 (0.675–1.523)	0.948	–	–
TNM staging	2.331 (1.206–4.504)	0.012	1.755 (1.141–2.700)	0.011	2.242 (1.284–3.913)	0.005
Blood vessel invasion	–	–	1.326 (0.793–2.217)	0.282	3.424 (1.828–6.414)	<0.001
HER-2 expression	7.566 (3.909–14.644)	<0.001	1.613 (1.008–2.579)	0.046	2.844 (1.117–7.241)	0.028
Histological grade	3.099 (1.557–6.169)	0.001	1.018 (0.685–1.513)	0.929	3.460 (1.754–6.828)	<0.001
SLN metastasis	–	–	5.875 (2.914–11.844)	<0.001	4.650 (2.041–10.595)	<0.001
ER	0.220 (0.113–0.430)	<0.001	1.281 (0.855–1.920)	0.23	0.329 (0.173–0.625)	0.001
PR	0.179 (0.091–0.354)	<0.001	0.350 (0.230–0.531)	<0.001	0.649 (0.361–1.165)	0.147

Note: –, no value.

## Discussion

With an estimated global incidence rate of 1.6 million annually, breast cancer is the most frequent malignancy in females [21]. Although its mortality rate has reduced since 1990, the fact that clinical characteristics of breast cancer patients vary from one to another based on their age, tumor grade, tumor stage, ER and PR status, and other noncancer-related factors posed a great challenge to seek biomarkers or combined markers for the improvement of diagnosis, prognosis, and prediction of breast cancer [22–25]. The present study investigated the link between HER-2 protein expression and SLN metastasis and prognosis in breast cancer in order to examine whether HER-2 could be used as a possible biomarker in identifying breast cancer or not.

The present study found that HER-2 expression was significantly higher in breast cancer tissues compared with adjacent tissues, which was positively correlated with SLN metastasis of breast cancer. These results suggested that higher expression of HER-2 promoted SLN metastasis in breast cancer. HER-2 is a cancer-related antigen of EGFR family that is involved in the tumorigenesis and tumor invasion [26]. It plays a key role in cell survival, apoptosis, aggression and differentiation, and the overexpression of HER-2 protein results in cellular malignant transformation in many kinds of tumors, including breast cancer, prostate cancer, ovarian cancer etc. [15]. HER-2 has been found to be overexpressed in 15–20% of primary breast cancers and related to worse prognosis and rising possibility of lymph node metastasis [27,28]. Cabioglu et al. [29] also pointed out that HER-2 overexpression might be a biological signature in predicting the SLN metastasis in patients with small breast cancers. Furthermore, it was shown that there was a close correlation between HER-2 overexpression and nodal metastasis because of the boost of HER-2 on cell proliferation, migration, and metastasis in breast cancer [30]. In addition, the present study showed that the expression of HER2 in breast cancer tissues was correlated with the expression levels of ER and PR. ER and PR are considered to be predictive markers for the patient response to hormonal therapy in breast cancer [31]. A previous study has demonstrated that changes in HER2 coexist with other prognostic factors such as the absence of ER and PR [32]. Moreover, the data show that the expression of ER and PR is highly associated between the breast cancer and SLN metastasis [33]. The molecular mechanism involved in the tumor metastasis to SLN remains relatively poorly understood, but it has been proved that the expression of vascular endothelial growth factor C (VEGF-C) is a potential pathogenesis [34]. VEGF-C, a ligand of VEGF receptor-3 (VEGFR-3), is a tyrosine kinase receptor that is expressed mostly in lymphatic endotheliocytes [35]. It is a key factor that promotes lymph node metastasis of tumor cells by increasing lymphangiogenesis in the SLN in a variety of cancers, including breast cancer [36]. It was demonstrated that there was a correlation between VEGF-C overexpression and the increased SLN risk in breast cancer patients [37,38]. Besides, HER-2 overexpression was proved to be correlated with the increased expression of VEGF-C in breast cancer, indicating that HER-2 was a mediator of SLN [39].

The present study also demonstrated that median DFS and OS were higher in patients with negative HER-2 expression than in patients with positive HER-2 expression, indicating that a higher HER-2 protein expression meant shorter survival rate. Multivariate analysis further showed that SLN metastasis and HER-2 expression were independent factors of DFS and OS of breast cancer patients. The results indicated that HER-2 was an important biomarker in the prognosis of breast cancer patients. HER-2 overexpression has been proved to be a strong biochemical marker for breast cancer prognosis [40]. It has also been proved that tumors with positive expression of EGFR protein had a significantly shorter survival time, suggesting a negative correlation of HER-2 protein expression with patients’ survival [41]. In addition, it was further revealed that the survival was worse and the overall relapse time was shorter in patients with positive HER-2 expression and lymph node metastasis than in patients with negative HER-2 expression and without lymph node metastasis [39]. Thus, the up-regulation of HER-2 was linked to a poorer prognosis in breast cancer [42]. Due to the great significance in the prediction and prognosis of breast cancer, HER-2 status has now become an important index for the evaluation of breast cancer patients [43].

In summary, the present study revealed that high expression of HER-2 was associated with SLN metastasis and poorer prognosis of breast cancer. It indicated that HER-2 might be a potential biomarker in the diagnosis and prognosis of breast cancer, laying a foundation for future HER-2-targeted therapy for breast cancer. Because the patients selected in the present study were all from Ningbo Yinzhou No. 2 Hospital, so the results can only represent the conditions in local area and cannot be applicable to patients in other areas. Besides, as a large number of previous researches focusing on the correlation of HER-2 with the prognosis of breast cancer have been carried out, it poses a great challenge for us to make innovation in this field. Moreover, due to small sample size in the present study, the correlation value between HER-2 and SLN metastasis is 0.548, which is a moderate correlation value. We would enlarge sample size in future studies to examine the clinical value of HER-2 in breast cancer.

## Abbreviations

DAB: diaminobenzidine
DFS: disease-free survival
ECD: extracellular ligand-binding domain
EGFR: epidermal growth factor receptor
ER: estrogen receptor
GAPDH: glyceraldehyde-3-phosphate dehydrogenase
HE: hematoxylin–eosin
HER-2: human epidermal growth factor receptor-2
IHC: immunohistochemistry
OS: overall survival
PR: progesterone receptor
qRT-PCR: quantitative real-time polymerase chain reaction
SBR: Scarff–Bloom–Richardson
SLN: sentinel lymph node
SLNB: sentinel lymph node biopsy
SP: streptavidin–peroxidase
VEGF-C: vascular endothelial growth factor C

## References

[1] Jemal A., Bray F., Center M.M., Ferlay J., Ward E. and Forman D. (2011) Global cancer statistics. CA Cancer J. Clin. 61, 69–90 21296855 10.3322/caac.20107

[2] Iwasaki M. and Tsugane S. (2011) Risk factors for breast cancer: epidemiological evidence from Japanese studies. Cancer Sci. 102, 1607–1614 21624009 10.1111/j.1349-7006.2011.01996.x

[3] Jia Y., Lu Y., Wu K., Lin Q., Shen W., Zhu M. (2013) Does night work increase the risk of breast cancer? A systematic review and meta-analysis of epidemiological studies Cancer Epidemiol. 37, 197–206 23403128 10.1016/j.canep.2013.01.005

[4] Zanghi G., Di Stefano G., Caponnetto A., Vecchio R., Lanaia A., La Terra A. (2014) Breast cancer and sentinel lymph node micrometastases: indications for lymphadenectomy and literature review. G. Chir. 35, 260–265 25644726 PMC4321503

[5] Carvalho S.M., Mourao Netto M., Lima E.N., Pimentel A.M., Makdissi F.B., Osorio C.A. (2010) Sentinel node biopsy in breast cancer: results in a large series. Braz. J. Med. Biol. Res. 43, 593–599 20512299 10.1590/s0100-879x2010007500048

[6] McArdle C.S., McMillan D.C., Greenlaw N. and Morrison D.S. (2010) Adjuvant radiotherapy and chemotherapy in breast cancer: 30 year follow-up of survival. BMC Cancer 10, 398 20673353 10.1186/1471-2407-10-398 PMC2918580

[7] Early Breast Cancer Trialists’Collaborative, G. (2005) Effects of chemotherapy and hormonal therapy for early breast cancer on recurrence and 15-year survival: an overview of the randomised trials. Lancet 365, 1687–1717 15894097 10.1016/S0140-6736(05)66544-0

[8] Li J., Zhang B.N., Fan J.H., Pang Y., Zhang P., Wang S.L. (2011) A nation-wide multicenter 10-year (1999-2008) retrospective clinical epidemiological study of female breast cancer in China. BMC Cancer 11, 364 21859480 10.1186/1471-2407-11-364 PMC3178543

[9] Guzzo F., Bellone S., Buza N., Hui P., Carrara L., Varughese J. (2012) HER2/neu as a potential target for immunotherapy in gynecologic carcinosarcomas. Int. J. Gynecol. Pathol. 31, 211–221 22498937 10.1097/PGP.0b013e31823bb24d PMC3366047

[10] Schrohl A.S., Pedersen H.C., Jensen S.S., Nielsen S.L. and Brunner N. (2011) Human epidermal growth factor receptor 2 (HER2) immunoreactivity: specificity of three pharmacodiagnostic antibodies. Histopathology 59, 975–983 22092409 10.1111/j.1365-2559.2011.04034.x PMC3263426

[11] Khademi B., Khademi B., Ghaderi A., Hosseini S.F. and Niknejad N. (2013) Early detection of serum levels of HER-2 in patients with head and neck squamous cell carcinoma. Iran. J. Otorhinolaryngol. 25, 161–168 24303437 PMC3846229

[12] He C., Bian X.Y., Ni X.Z., Shen D.P., Shen Y.Y., Liu H. (2013) Correlation of human epidermal growth factor receptor 2 expression with clinicopathological characteristics and prognosis in gastric cancer. World J. Gastroenterol. 19, 2171–2178 23599643 10.3748/wjg.v19.i14.2171 PMC3627881

[13] Kaur A. and Dasanu C.A. (2011) Targeting the HER2 pathway for the therapy of lower esophageal and gastric adenocarcinoma. Expert. Opin. Pharmacother. 12, 2493–2503 21967344 10.1517/14656566.2011.605354

[14] Lambein K., Praet M., Forsyth R., Van den Broecke R., Braems G., Matthys B. (2011) Relationship between pathological features, HER2 protein expression and HER2 and CEP17 copy number in breast cancer: biological and methodological considerations. J. Clin. Pathol. 64, 200–207 21177747 10.1136/jcp.2010.084863

[15] Tai W., Mahato R. and Cheng K. (2010) The role of HER2 in cancer therapy and targeted drug delivery. J. Control Release 146, 264–275 20385184 10.1016/j.jconrel.2010.04.009 PMC2918695

[16] Meng X., Wang R., Huang Z., Zhang J., Feng R., Xu X. (2014) Human epidermal growth factor receptor-2 expression in locally advanced rectal cancer: association with response to neoadjuvant therapy and prognosis. Cancer Sci. 105, 818–824 24730770 10.1111/cas.12421 PMC4317932

[17] Benson J.R., Weaver D.L., Mittra I. and Hayashi M. (2003) The TNM staging system and breast cancer. Lancet Oncol. 4, 56–60 12517540 10.1016/s1470-2045(03)00961-6

[18] Bansal C., Singh U.S., Misra S., Sharma K.L., Tiwari V. and Srivastava A.N. (2012) Comparative evaluation of the modified Scarff-Bloom-Richardson grading system on breast carcinoma aspirates and histopathology. Cytojournal 9, 4 22363393 10.4103/1742-6413.92550 PMC3280007

[19] Zhao S., Xu L., Liu W., Lv C., Zhang K., Gao H. (2015) Comparison of the expression of prognostic biomarkers between primary tumor and axillary lymph node metastases in breast cancer. Int. J. Clin. Exp. Pathol. 8, 5744–5748 26191291 PMC4503162

[20] Bakhtiar N., Jaleel F., Moosa F.A., Qureshi N.A. and Jawaid M. (2016) Sentinel lymph node identification by blue dye in patients with breast carcinoma. Pak. J. Med. Sci. 32, 448–451 27182259 10.12669/pjms.322.9563 PMC4859042

[21] Cuzick J., Sestak I., Cawthorn S., Hamed H., Holli K., Howell A. (2015) Tamoxifen for prevention of breast cancer: extended long-term follow-up of the IBIS-I breast cancer prevention trial. Lancet Oncol. 16, 67–75 25497694 10.1016/S1470-2045(14)71171-4 PMC4772450

[22] Patnaik J.L., Byers T., DiGuiseppi C., Dabelea D. and Denberg T.D. (2011) Cardiovascular disease competes with breast cancer as the leading cause of death for older females diagnosed with breast cancer: a retrospective cohort study. Breast Cancer Res. 13, R64 21689398 10.1186/bcr2901 PMC3218953

[23] Andorfer C.A., Necela B.M., Thompson E.A. and Perez E.A. (2011) MicroRNA signatures: clinical biomarkers for the diagnosis and treatment of breast cancer. Trends Mol. Med. 17, 313–319 21376668 10.1016/j.molmed.2011.01.006

[24] McKenzie F. and Jeffreys M. (2009) Do lifestyle or social factors explain ethnic/racial inequalities in breast cancer survival. Epidemiol. Rev. 31, 52–66 19675112 10.1093/epirev/mxp007

[25] Dunnwald L.K., Rossing M.A. and Li C.I. (2007) Hormone receptor status, tumor characteristics, and prognosis: a prospective cohort of breast cancer patients. Breast Cancer Res. 9, R6 17239243 10.1186/bcr1639 PMC1851385

[26] Carmichael M.G., Benavides L.C., Holmes J.P., Gates J.D., Mittendorf E.A., Ponniah S. (2010) Results of the first phase 1 clinical trial of the HER-2/neu peptide (GP2) vaccine in disease-free breast cancer patients: United States Military Cancer Institute Clinical Trials Group Study I-04. Cancer 116, 292–301 19924797 10.1002/cncr.24756

[27] Wolff A.C., Hammond M.E., Hicks D.G., Dowsett M., McShane L.M., Allison K.H. (2013) Recommendations for human epidermal growth factor receptor 2 testing in breast cancer: American Society of Clinical Oncology/College of American Pathologists clinical practice guideline update. J. Clin. Oncol. 31, 3997–4013 24101045 10.1200/JCO.2013.50.9984

[28] Duran M.C., Vega F., Moreno-Bueno G., Artiga M.J., Sanchez L., Palacios J. (2008) Characterisation of tumoral markers correlated with ErbB2 (HER2/Neu) overexpression and metastasis in breast cancer. Proteomics Clin. Appl. 2, 1313–1326 21136925 10.1002/prca.200780020

[29] Cabioglu N., Yazici M.S., Arun B., Broglio K.R., Hortobagyi G.N., Price J.E. (2005) CCR7 and CXCR4 as novel biomarkers predicting axillary lymph node metastasis in T1 breast cancer. Clin. Cancer Res. 11, 5686–5693 16115904 10.1158/1078-0432.CCR-05-0014

[30] Bartlett J.M., Ellis I.O., Dowsett M., Mallon E.A., Cameron D.A., Johnston S. (2007) Human epidermal growth factor receptor 2 status correlates with lymph node involvement in patients with estrogen receptor (ER) negative, but with grade in those with ER-positive early-stage breast cancer suitable for cytotoxic chemotherapy. J. Clin. Oncol. 25, 4423–4430 17906205 10.1200/JCO.2007.11.0973

[31] Hammond M.E., Hayes D.F., Wolff A.C., Mangu P.B. and Temin S. (2010) American society of clinical oncology/college of american pathologists guideline recommendations for immunohistochemical testing of estrogen and progesterone receptors in breast cancer. J. Oncol. Pract. 6, 195–197 21037871 10.1200/JOP.777003 PMC2900870

[32] Badowska-Kozakiewicz A.M., Sobol M., Patera J. and Kozlowski W. (2013) Immunohistochemical evaluation of human epidermal growth factor receptor 2 and estrogen and progesterone receptors in invasive breast cancer in women. Arch. Med. Sci. 9, 466–471 23847668 10.5114/aoms.2012.31010 PMC3701965

[33] Desouki M.M., Atta I.S., Wolff D.J. and Self S.E. (2016) Comparison between HER2, Estrogen receptors and progesterone receptors in primary breast carcinomas and matched lymph node metastases. Turk. Patoloji. Derg. 32, 178–185 27562392 10.5146/tjpath.2015.01354

[34] Hirakawa S., Brown L.F., Kodama S., Paavonen K., Alitalo K. and Detmar M. (2007) VEGF-C-induced lymphangiogenesis in sentinel lymph nodes promotes tumor metastasis to distant sites. Blood 109, 1010–1017 17032920 10.1182/blood-2006-05-021758 PMC1785149

[35] Onogawa S., Kitadai Y., Tanaka S., Kuwai T., Kuroda T. and Chayama K. (2004) Regulation of vascular endothelial growth factor (VEGF)-C and VEGF-D expression by the organ microenvironment in human colon carcinoma. Eur. J. Cancer 40, 1604–1609 15196547 10.1016/j.ejca.2004.02.026

[36] Guo B., Zhang Y., Luo G., Li L. and Zhang J. (2009) Lentivirus-mediated small interfering RNA targeting VEGF-C inhibited tumor lymphangiogenesis and growth in breast carcinoma. Anat. Rec. (Hoboken) 292, 633–639 19382240 10.1002/ar.20893

[37] Dadras S.S., Lange-Asschenfeldt B., Velasco P., Nguyen L., Vora A., Muzikansky A. (2005) Tumor lymphangiogenesis predicts melanoma metastasis to sentinel lymph nodes. Mod. Pathol. 18, 1232–1242 15803182 10.1038/modpathol.3800410

[38] Mylona E., Alexandrou P., Mpakali A., Giannopoulou I., Liapis G., Markaki S. (2007) Clinicopathological and prognostic significance of vascular endothelial growth factors (VEGF)-C and -D and VEGF receptor 3 in invasive breast carcinoma. Eur. J. Surg. Oncol. 33, 294–300 17129704 10.1016/j.ejso.2006.10.015

[39] Nathanson S.D., Slater R., Debruyn D., Kapke A. and Linden M. (2006) Her-2/neu expression in primary breast cancer with sentinel lymph node metastasis. Ann. Surg. Oncol. 13, 205–213 16408141 10.1245/ASO.2006.03.032

[40] Ivkovic-Kapicl T., Knezevic-Usaj S., Djilas-Ivanovic D. and Panjkovic M. (2007) Correlation of HER-2/neu protein overexpression with other prognostic and predictive factors in invasive ductal breast cancer. In Vivo 21, 673–678 17708365

[41] Ludovini V., Bellezza G., Pistola L., Bianconi F., Di Carlo L., Sidoni A. (2009) High coexpression of both insulin-like growth factor receptor-1 (IGFR-1) and epidermal growth factor receptor (EGFR) is associated with shorter disease-free survival in resected non-small-cell lung cancer patients. Ann. Oncol. 20, 842–849 19153117 10.1093/annonc/mdn727

[42] Esteva F.J., Sahin A.A., Smith T.L., Yang Y., Pusztai L., Nahta R. (2004) Prognostic significance of phosphorylated P38 mitogen-activated protein kinase and HER-2 expression in lymph node-positive breast carcinoma. Cancer 100, 499–506 14745865 10.1002/cncr.11940

[43] Moelans C.B., de Weger R.A., van Blokland M.T., Ezendam C., Elshof S., Tilanus M.G. (2009) HER-2/neu amplification testing in breast cancer by multiplex ligation-dependent probe amplification in comparison with immunohistochemistry and in situ hybridization. Cell Oncol. 31, 1–10 19096145 10.3233/CLO-2009-0461 PMC4618800

